# Current challenges for confronting the public health problem of snakebite envenoming in Central America

**DOI:** 10.1186/1678-9199-20-7

**Published:** 2014-03-06

**Authors:** José María Gutiérrez

**Affiliations:** 1 Instituto Clodomiro Picado, Facultad de Microbiología, Universidad de Costa Rica, San José, Costa Rica

**Keywords:** Central America, Snakebite, Envenoming, Antivenoms, Public health

## Abstract

Snakebite envenoming is a serious public health problem in Central America, where approximately 5,500 cases occur every year. Panama has the highest incidence and El Salvador the lowest. The majority, and most severe, cases are inflicted by the pit viper *Bothrops asper* (family Viperidae), locally known as ‘terciopelo’, ‘barba amarilla’ or ‘equis’. About 1% of the bites are caused by coral snakes of the genus *Micrurus* (family Elapidae). Despite significant and successful efforts in Central America regarding snakebite envenomings in the areas of research, antivenom manufacture and quality control, training of health professionals in the diagnosis and clinical management of bites, and prevention of snakebites, much remains to be done in order to further reduce the impact of this medical condition. This essay presents seven challenges for improving the confrontation of snakebite envenoming in Central America. Overcoming these challenges demands a coordinated partnership of highly diverse stakeholders though inter-sectorial and inter-programmatic interventions.

## Review

### Introduction

**Figure 1:**
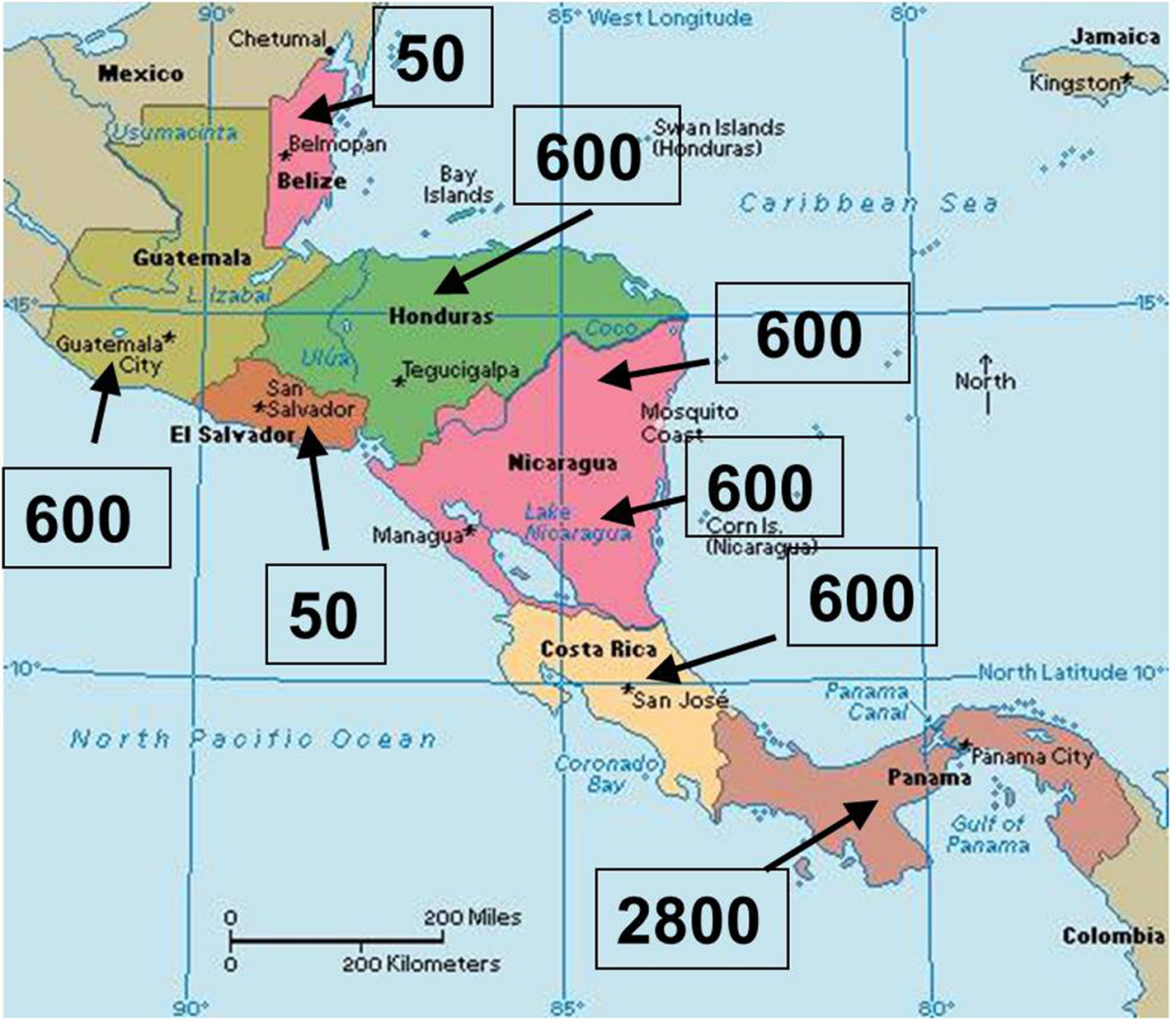
**Estimated absolute number of snakebite cases in the various Central American countries.** These numbers are based on hospital data. Incidences are presented in Table 1.

**Figure 2:**
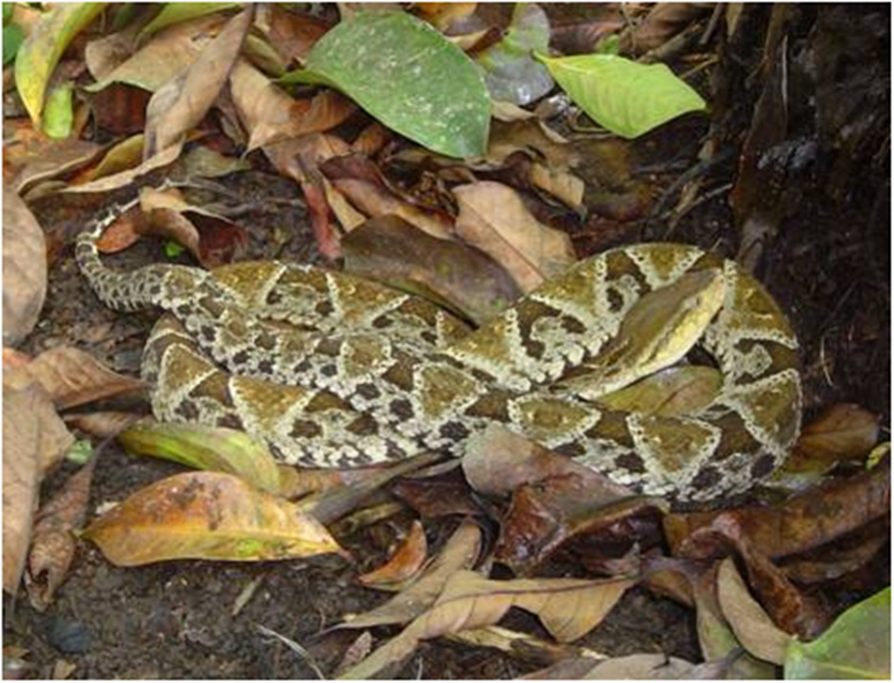
***Bothrops asper *(family Viperidae) is the most important snake from the medical standpoint in Central America.** It is distributed in the humid tropical lowlands and provokes the majority of cases, and the most serious ones. It is also responsible for the majority of fatal cases in the region. This snake adapts very well to altered environments, such as agricultural fields and pastures. Photo by Mahmood Sasa and reprinted from “Confronting the neglected problem of snake bite envenoming: the need for a global partnership” by Gutiérrez *et al.*, *PLOS Medicine*, 2006, 3(6), e150 [7]. Creative Commons Attribution License (CCAL).

Snakebite envenoming constitutes a serious public health problem in Central America [1-5]. It has been estimated that around 5,500 snakebite cases reach health centers in the region (Figure 1), although it is likely that this represents an underreporting because, as occurs in some regions of Asia, Africa and other portions of Latin America, an unknown number of snake-bitten people are not treated at health facilities [4]. The absolute number of accidents, and the corresponding incidence, varies within the region. Panama has the highest total number of cases, and its incidence is the highest not only in Central America, but also in Latin America as a whole (Table 1). In contrast, the lowest incidence is found in El Salvador. It is likely that such variations are related to the exposure of human populations to the species *Bothrops asper*, medically the most important snake in the region (Figure 2). In Panama this pit viper is widely distributed in centers of high human population density, whereas in El Salvador, located in the dry Pacific corridor of the region, *B. asper* is absent [6].

**Table 1:** Estimated total number of cases per year and incidence of snakebites in the various countries of Central America

**Country**	**Total number of cases per year**	**Incidence (per 100,000 inhabitants per year)**
Guatemala	600	4.2
Belize	50	15.2
Honduras	600	7.2
El Salvador	50	0.8
Nicaragua	600	10.5
Costa Rica	600	12.9
Panama	2,800	79.8

The vast majority of the abundant snake species in Central America are non-venomous (families Typhlopidae, Leptotyphlopidae, Anomalepidae, Boidae, Loxocemidae and Ungaliophidae) [8-10]. Species classified in the family Colubridae (*sensu lato*) include snakes with venom glands and a fang system located in the posterior part of the maxilla. From the biological standpoint these are venomous snakes; however, they rarely bite humans, owing mostly to the difficulty with which they inject their toxic secretions, and also to the low intrinsic toxicity of some of these venoms. Hence, signs and symptoms of bites by colubrids in Central America do not exceed local edema and bruising [4,11]. In contrast, envenomings that might be life-threatening are inflicted by snakes classified in the families Elapidae (yellow-bellied sea snake and coral snakes) and Viperidae (pit vipers). In the latter, besides mortality, one serious consequence of envenomings is the development of permanent sequelae associated with tissue loss and dysfunction.

Envenomings by pit vipers, among which the most serious are inflicted by *Bothrops asper*, locally known as ‘terciopelo’, ‘barba amarilla’ or ‘equis’ (Figure 2), are characterized by prominent tissue damage at the bite site involving pain, edema, blistering, hemorrhage and necrosis of soft tissues [4,12,13]. In moderate and severe cases, systemic manifestations occur, characterized mostly by bleeding, coagulopathy, acute kidney injury, and cardiovascular shock [4,12,13]. On the other hand, envenomings by coral snakes (genus *Micrurus*) present neurotoxic manifestations provoked by a neuromuscular blockage predominantly induced by post-synaptically acting low-molecular-mass neurotoxins [4]. Although there are no well-described cases of envenomings by the yellow-bellied sea snake (*Pelamis platurus*, family Elapidae), it is likely that they would be associated with neurotoxic features similar to envenomings by coral snakes [4].

The severity of snakebite envenomings depends on several factors, namely:

•The volume of venom injected.

•The anatomical site of injection (bites on the head and trunk tend to be more complicated than those on the feet or hands).

•The size and physiological condition of the victim (bites in children tend to be more serious than in adults).

•Time elapsed between the bite and medical attention.

The only scientifically validated treatment for snakebite envenoming is the administration of safe and effective animal-derived antivenoms [14]. Antivenoms for pit vipers and coral snakes are manufactured by Instituto Clodomiro Picado, Universidad de Costa Rica, and distributed to all Central American countries. In addition, other antivenoms, manufactured in Mexico and Argentina, are also available in the region.

Despite the fact that this problem is attended by the public health systems in the region, with the consequent reduction of mortality in the last decades (see for example Rojas *et al*. [15]), a number of challenges remain in Central America for improving the manner of understanding and confronting this health issue. The present work discusses some of these challenges and presents some proposals for improving the analysis, prevention and treatment of snakebite envenomings in Central America.

### Challenge 1: To improve the knowledge on the epidemiology and the physical, social and psychological consequences of snakebite envenomings

There are various studies published on the epidemiology and mortality of snakebite envenoming in Central America, especially in Costa Rica [1,15-18]. Likewise, Panamá and Nicaragua have developed epidemiological surveillance systems that have provided valuable information on the incidence and mortality of snakebites in these countries (Hildaura Acosta and Luz Marina Lozano, personal communications). Nevertheless, no systematic information is available on this subject in relation to Honduras, Guatemala, El Salvador and Belize. Hence, there is an urgent need to undertake coordinated regional efforts to gather information on the epidemiological parameters associated with this condition. It is suggested that a concerted regional effort, coordinated by the health ministries in the region and with the support of the Pan American Health Organization (PAHO), should be promoted to achieve this goal. Once implemented, such a system will provide updated information of crucial importance for promoting a knowledge-based system of decision-making on this subject. Compulsory reporting of snakebite envenomings has been introduced in some countries, and needs to be generalized to the rest of the region.

In addition to the provision of data on incidence and mortality, this information will allow the identification of vulnerable regions in each country, particularly with regard to vulnerable sectors, such as the indigenous groups. Moreover, there is a need to develop studies of community-based and household surveys that could provide a more realistic view of the magnitude of the problem. This type of research has been undertaken in other parts of the world, such as Bangladesh and India, where it showed that the magnitude of this problem is much larger than what is reflected by hospital-based statistics [19,20]. Finally, there is an urgent need to gather information on the incidence of permanent sequelae in people suffering from snakebite envenoming. This is a highly neglected aspect of the issue, since people that survive envenomings usually return to their communities without following up on the physical and psychological consequences of these accidents, and their quality-of-life impact on.

The effect of this disease in specific groups – including children, indigenous communities, agricultural workers etc. – should be investigated in order to discern the particular aspects in which such groups are affected. The incorporation of social research for understanding snakebite envenomings and their implications is of paramount relevance owing to the impact of cultural, sociological, economical and psychological variables in the overall picture of this condition [21]. Clearly, research on public health and other social aspects of snakebite envenoming in the region is badly needed, through concerted efforts of ministries of health, research groups in universities and other institutions, and organizations of the civil society.

### Challenge 2: To improve the knowledge on the clinical manifestations of envenomings and the response to antivenom treatment

Since the pioneering work of Clodomiro Picado Twight [22], a large body of scientific knowledge has been accumulated on the biology of the venomous snakes of Central America (see, for example, [1,6,8-10] and references therein), and on the composition and action mechanism of snake venoms (see, for example, the special issue of *Toxicon* devoted to *Bothrops asper* in 2009 [23] and a recent review by Lomonte *et al.*[24] on the proteomics of venoms of Central American snakes). Moreover, the technological innovations and improvements in antivenom manufacturing have received attention by researchers in the region, as well as the analysis of the preclinical neutralizing efficacy of antivenoms against the venoms of Central American snakes (see reviews [25-27] and references therein). In contrast, there is relatively little research published on the clinical aspects of envenomings and on the therapeutic response to antivenoms [28]. In fact, the most detailed analyses of the efficacy and safety, in the clinical setting, of antivenoms manufactured in Costa Rica have been performed in Colombia, through the studies of Rafael Otero-Patiño and colleagues [29-32].

There is a need to characterize the clinical manifestations of envenomings induced by various snakes in the region, with the aim of identifying clinical patterns characteristic of some species. Some pending questions in this area are:

•Do envenomings by newborn/neonate and juvenile pit vipers differ from those provoked by adult specimens, as suggested by their different proteomic and toxinological profiles?

•Can bites by *Bothriechis* sp. species induce envenomings with systemic manifestations and, if so, what are these systemic effects?

•What is the clinical picture that characterizes envenomings by terrestrial species of the genera *Porthidium*, *Cerrophidium* and *Atropoides*, which are endemic to Central America?

•Are envenomings by *Lachesis stenophrys* and *L. melanocephala* characterized by the unique vagal-type effect described in cases by South American bushmasters?

•Are envenomings by *Micrurus* sp. characterized only by neurotoxic manifestations, or do some species provoke other effects, such as those described in bites by some South American coral snakes?

Moreover, the therapeutic effect of antivenoms used in the region in envenomings by different pit viper species has to be investigated in the clinical setting. It is not clear, for instance, whether the polyspecific antivenom is effective, at the clinical level, for neutralizing the venom of *Agkistrodon bilineatus*, although preclinical results may suggest so [33]. Thus, there is a need to develop partnerships between university research groups and clinicians to improve the knowledge on snakebite envenomings and their treatment. In this aspect, the efforts being carried out in Panama, where strong links have been established between researchers at the University of Panamá and a network of clinicians in various hospitals, constitutes a highly promising step forward (Hildaura Acosta, personal communication). It is expected that such efforts will bring novel and relevant information in the coming years.

### Challenge 3: To improve the availability and accessibility of antivenoms in all Central American countries

Antivenoms for the treatment of pit viper and coral snakebite envenomings have been produced in Costa Rica since 1967, an effort consolidated by the creation of Instituto Clodomiro Picado in 1970 [34,35]. In addition, other manufacturers in Latin America produce antivenoms that are effective in the treatment of these envenomings in Central America. Such regional capacity to provide antivenoms has contributed to antivenom availability in this region. Moreover, the health ministries and other institutions of the public health system (such as the social security systems) have developed purchasing schemes to obtain these products on a regular basis. Nevertheless, due to budget constraints, the amount of antivenom purchased by some countries is insufficient to guarantee complete coverage of all antivenom needs. On top of this, the distribution of antivenoms within each country poses difficult challenges for the public health systems. The lack of adequate epidemiological information on snakebites in some countries does not allow for developing knowledge-based antivenom distribution schemes. Consequently, antivenoms are often scarce at health clinics in some rural areas where snakebites are frequent, which has an impact on the evolution of the cases, since patients need to be transported to clinics or hospitals in other locations, with a consequent delay in the onset of treatment.

**Figure 3:**
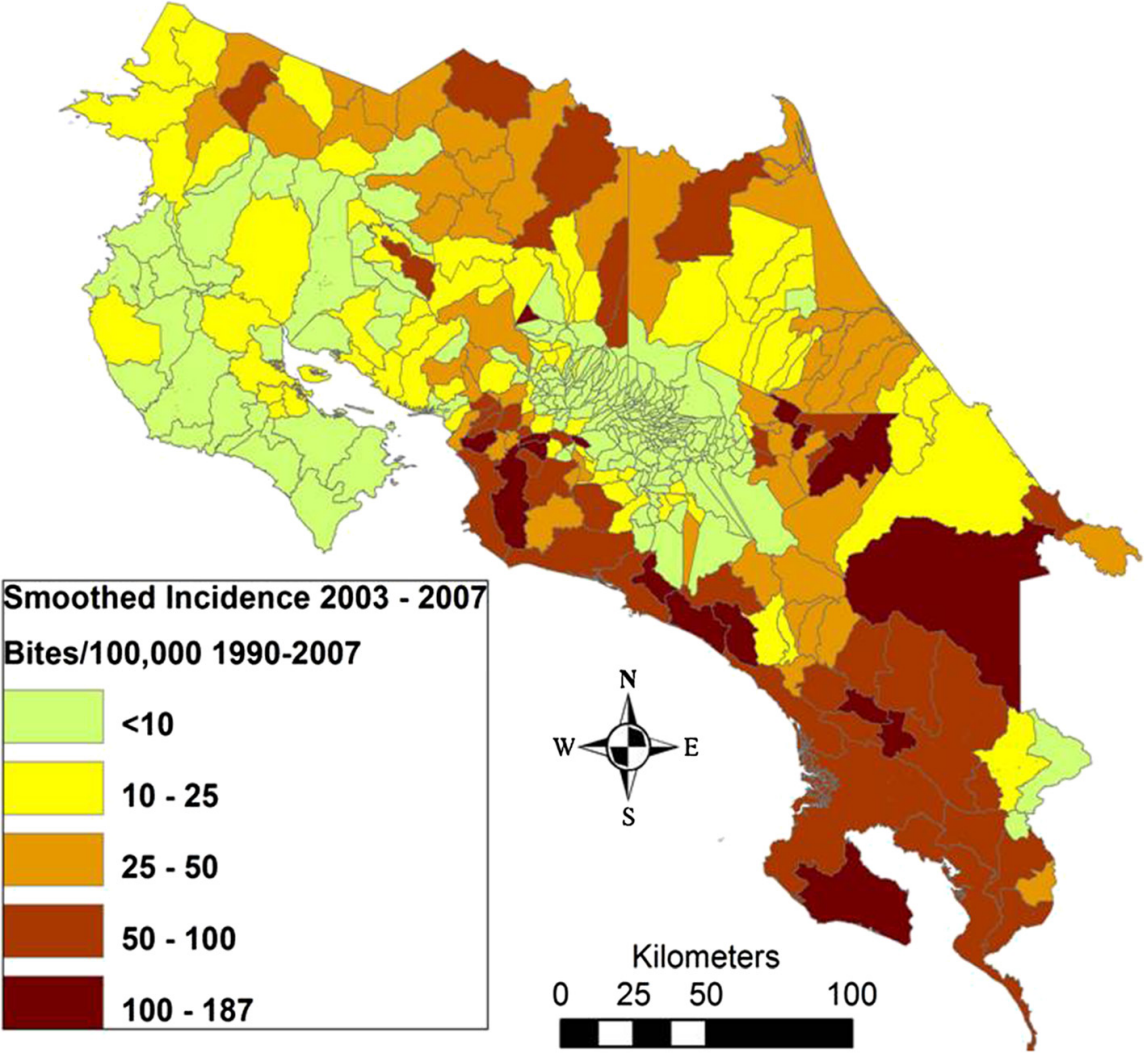
**Incidence of snakebite in Costa Rica per district per 100,000 population (1990-2007).** Prepared by Erik Hansson and reprinted from “Using geographical information systems to identify populations in need of improved accessibility to antivenom treatment for snakebite envenoming in Costa Rica” by Hansson *et al.*, *PLOS Neglected Tropical Diseases*, 2013, 7 (1), e2009 [37]. Creative Commons Attribution License (CCAL).

**Figure 4:**
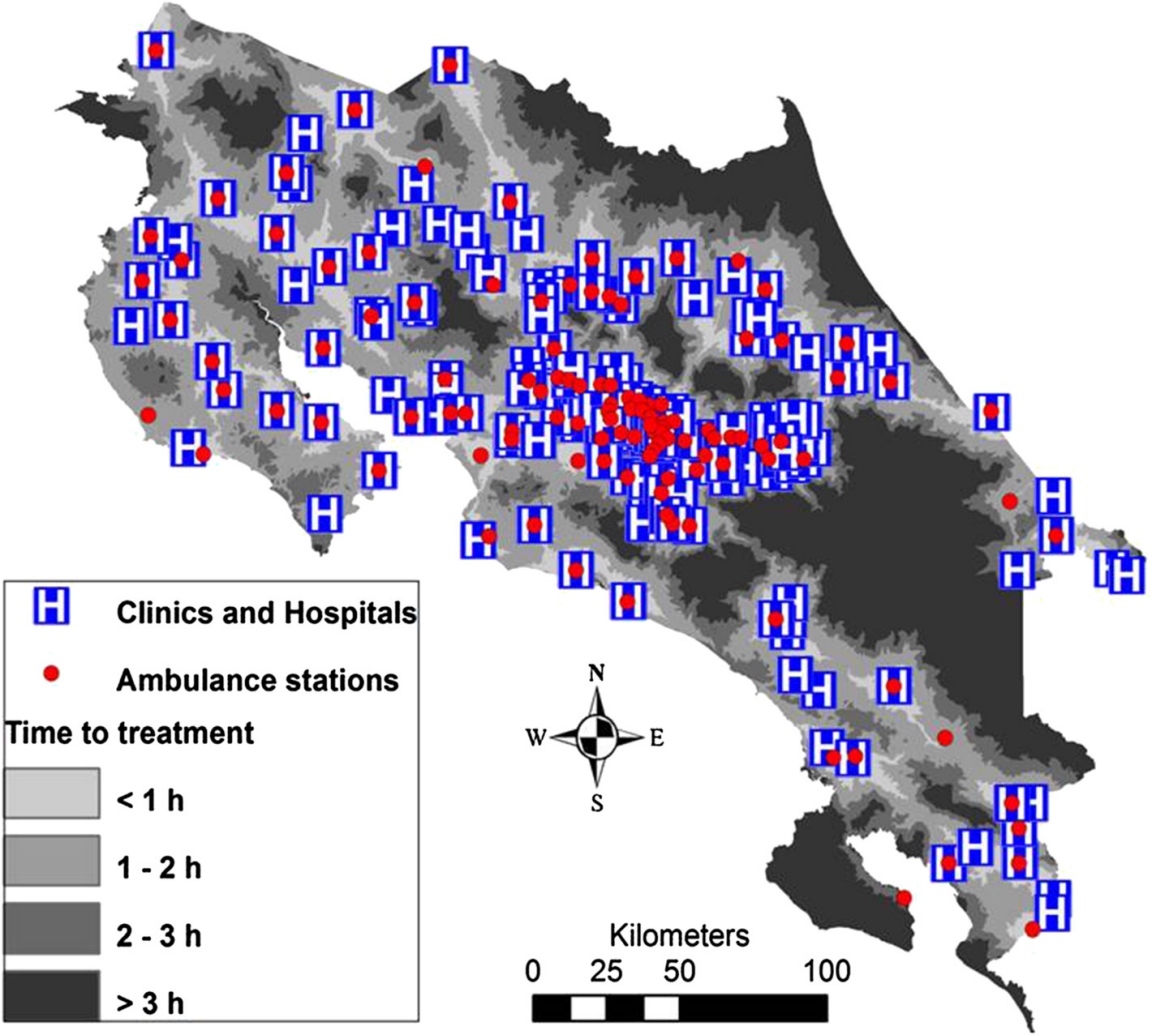
**Distribution of hospitals, clinics and ambulance stations in Costa Rica, and estimated time to reach hospitals or clinics in various regions of the country.** As shown in Figure 3, regions where transportation to health facilities takes longer correspond to regions of high snakebite incidence. Prepared by Erik Hansson and reprinted from Hansson *et al.*[37].

A relevant challenge in Central America is the buildup of a solid epidemiological base through which the health institutions can design a knowledge-based platform for antivenom distribution. The use of geographical information system (GIS) tools should help to identify regions of high vulnerability to snakebites which require improvements in antivenom deployment (see for example [36,37] for GIS studies performed in Nicaragua and Costa Rica) (Figures 3 and 4). Particular attention must be given to identifying vulnerable regions and groups, such as indigenous communities and other social and ethnic groups largely deprived of health services. The analysis of snakebite cases should be performed *vis-à-vis* the distribution of health services, and the distances between communities and nearest health clinics (see for instance [37] for the case of Costa Rica) (Figure 4).

In regions of high snakebite incidence where hospitals or clinics are absent, the allocation of antivenoms to health posts at the primary-care level should be carefully considered, through integrated programs ensuring maintenance of cold chain conditions, appropriate training of health staff, and provision of antivenoms and other drugs (including adrenaline, antihistamines and steroids) required for the management of envenomings. In this regard, and as a matter of example, proposals for snakebite management on an integrated national basis have been presented in Colombia [38] and in Nigeria [39]. A key aspect in these proposals has to do with the allocation of antivenoms and qualified staff to primary-care health facilities, thus ensuring a rapid administration of antivenom to bitten victims. Depending on the severity and complications of the case, patients could then be transferred to regional clinics or hospitals. In Costa Rica, for instance, antivenoms are distributed to health facilities at various levels of specialization, such as hospitals, clinics and some centers at the primary-care level (EBAIS – basic teams of integrated health attention) [40]. This has contributed to a reduction in the time lapse between the bite and the provision of antivenom.

### Challenge 4: To ensure the adequate clinical use of antivenoms

Effective management of snakebite envenomings at the clinical level demands a proper knowledge on the main aspects of their clinical manifestations and complications, diagnosis, and treatment, not only regarding the use of antivenom (including the management of adverse reactions), but also the ancillary therapy of the cases. The acquisition of this knowledge by health staff requires concerted actions at various levels, such as university courses in Medicine and Nursing schools, and permanent education programs, especially for physicians, nurses and other health staff working in areas of high incidence of envenomings.

The preparation of national and regional guidelines is of utmost relevance, in order to ensure a standard management of these cases with a solid scientific basis. Significant advances have been made in Panamá (Hildaura Acosta, personal communication) and Costa Rica, where snakebite management protocols and algorithms have been prepared and where permanent teaching activities take place at universities and health facilities. Seminars and related activities on the subject are regularly organized in some countries in the region, but in others there remains a need to develop permanent training programs; this demands concerted national and regional efforts. In this regard, the use of communication and information technologies, such as those designed for videoconferences, should be promoted. One example is the PAHO-supported platform Elluminate, which has been used for training activities on this subject in Honduras, Costa Rica and Panamá.

### Challenge 5: To provide attention and support to people suffering from sequelae of snakebite envenomings

One of the most neglected aspects of snakebite envenoming is following up on accident victims who develop permanent physical or psychological sequelae, especially those resulting from cases of viperid snakebites. The actual incidence of these sequelae is largely unknown, because survivors leave the hospital and follow-up of these cases is not performed. Since these accidents mostly affect poor and vulnerable people living in rural areas, the impact in their quality of life is likely to be dramatic. On one hand, this greatly affects their physical integrity and, consequently, their ability to perform field work, thus contributing to the vicious cycle of poverty. In addition, the psychological consequences of such a traumatic event are likely to be high, as demonstrated in a study on snakebite victims performed in Sri Lanka [41].

There is a pressing need to study the magnitude and characteristics of this problem in the region through integrated and interdisciplinary research projects. The relevance of social research in this aspect of the snakebite problem cannot be overestimated. The information generated in such studies will serve as the basis for the development of interventions by governmental and non-governmental groups with the aim of alleviating the long-term consequences of envenomings. The examples in which non-governmental organizations of the civil society have been created to improve the management of many diseases and mitigate their impact on the affected population, should serve to stimulate not only learning but also the development of similar organizations in support of snakebite victims.

### Challenge 6: To improve the prevention of snakebites

**Figure 5:**
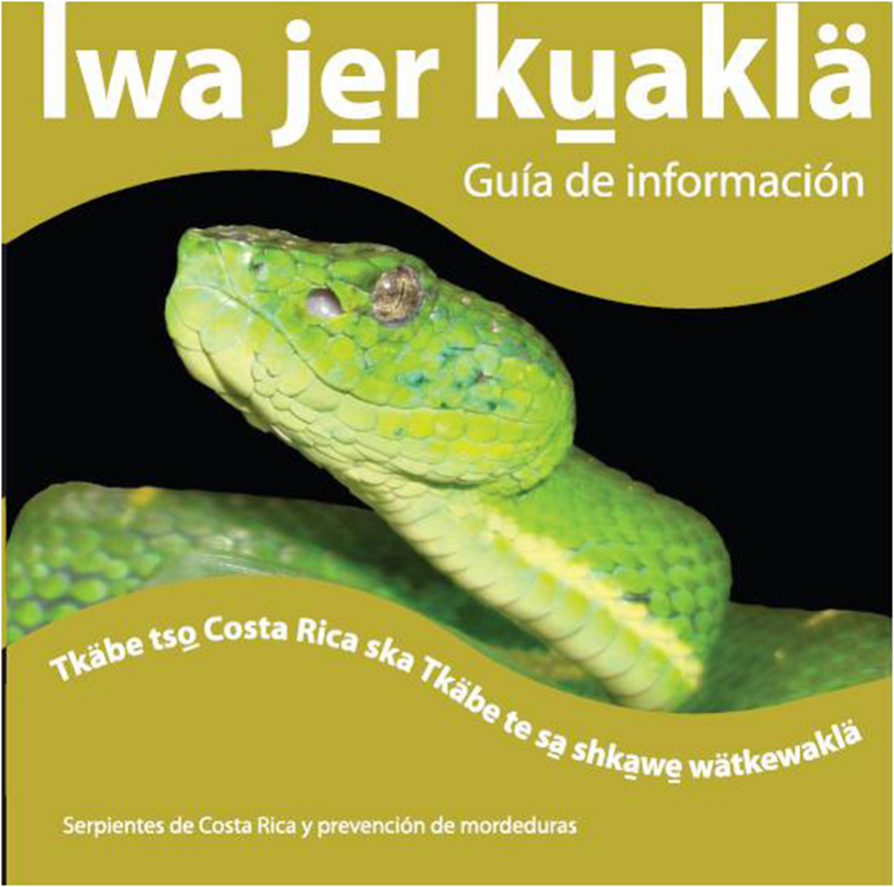
**Cover of a publication used in education campaigns to promote the prevention of snakebites in indigenous communities of the Cabécar ethnic group in Costa Rica.** This material was prepared in the Spanish and Cabécar languages by a cooperative project coordinated by Dr. Laura Monturiol (Instituto Clodomiro Picado) and developed by Instituto Clodomiro Picado (Universidad de Costa Rica), Caja Costarricense del Seguro Social, the Ministry of Education, and the Cabécar people. It was widely distributed in the Cabécar communities and used in local schools as part of a prevention campaign [42]. *Serpientes de Costa Rica y prevención de mordeduras: Guía de información*, 2009, Instituto Clodomiro Picado, Facultad de Microbiología, Universidad de Costa Rica. Reprinted approved by the General Director of the Institute.

Information gathered on the epidemiology of snakebite envenomings in Central America highlights several trends that are very helpful in designing effective prevention campaigns. The majority of accidents occur in the rural population in people devoted to agricultural work; hence, priority attention should be given to the provision of basic information on preventive measurements in these groups and communities. To this end, it is relevant to work in close coordination with local community organizations, in order to design the most appropriate intervention programs, which should be tailored to the specific cultural and social features of the populations. One important case to consider has to do with indigenous communities; appropriately designed campaigns must be prepared in local languages and should be adapted to the world view of these groups. In Costa Rica, for instance, Instituto Clodomiro Picado and Caja Costarricense del Seguro Social have developed a prevention program for the communities of the Cabécar ethnic group inhabiting the Caribbean region; this program was developed in close collaboration with the communities and with health and educational institutions in the region [42] (Figure 5).

Most of the snakebites in Central America occur on the feet (~50%) and hands (~30%) [1,4]. Therefore, the promotion of basic prevention measures should include the use of footwear and of sticks to test the ground before using the hands in agricultural duties. Moreover, using a torch after sunset and keeping the areas surrounding houses cleaned in regions of high abundance of snakes, especially *Bothrops asper*, are measures that reduce the likelihood of an accident. Finally, but not less important, is the community-based organization of rapid transportation of bitten people to the nearest health center. It is well known that the prognosis of an envenoming is directly related to the time lapse between the bite and the onset of treatment. Therefore, avoiding unnecessary delays due to the performance of ineffective and often harmful first aid interventions, and ensuring rapid transportation to the health post are key components of an adequate strategy to confront this problem. In Nepal, for instance, an effective motorcycle transportation system, together with community education campaigns, has proven highly effective at reducing the incidence and mortality due to snakebite envenomings [43].

### Challenge 7: To raise awareness of the relevance of snakebite envenoming in politicians and in the general population

The confrontation of snakebite envenoming as a public health problem on a global basis has been greatly hampered by its systematic neglect among public health and political authorities [7,44,45]. The fact that snakebite is not an infectious or parasitic disease has contributed to its alienation from the mainstream of international strategies to confront neglected tropical diseases [44,45]. Likewise, at the national level, a number of factors, such as poor epidemiological information, lack of a political voice representing the affected population, weak advocacy efforts by researchers, health promoters and community organizations, and little interest from the pharmaceutical sector, have largely contributed to the invisibility of this serious medical condition.

This scenario should be overcome by a concerted advocacy program at various levels. On a worldwide basis, one of the main goals of the Global Snakebite Initiative (GSI –http://www.snakebiteinitiative.org) is to promote the global awareness of the impact of snakebite envenomings [45]. Coordinated efforts have to be performed at regional and national levels as well. Of greatest relevance is the permanent communication with public health political authorities, mostly ministries of health, in order to upgrade the status of snakebite envenoming on the public health agenda. In Central America, this should result in the inclusion of discussions on snakebites at the regional meetings of ministers and in the design of coordinated actions in the region. To attain this goal, it is necessary to forge alliances between diverse groups, such as academics, health professionals, public health promoters and community-based organizations. It is only through the concerted efforts of such a wide array of stakeholders that this problem is going to receive proper attention. The inclusion of snakebite envenoming within the national and regional plans to combat neglected tropical diseases should be emphasized, by its incorporation in the packages designed for tropical infectious diseases, thus optimizing the use of resources [45].

## Conclusion

As observed in many regions of Africa, Asia, Latin America and Oceania, snakebite envenomings represent a serious public health problem in Central America. Although valuable efforts have been made in the region to perform research on snakes, venoms, and envenomings, to train health professionals and students in the details of the clinical diagnosis and management of envenomings, to produce and distribute safe and effective antivenoms to all the countries, and to promote the prevention of snakebites – much remains to be done in order to reduce the impact of this medical condition. The challenges discussed in this work present some of the issues that should be considered. Of highest relevance is the need to incorporate stakeholders from a wide spectrum of sectors in the study and solution of the problems associated with snakebites. It is only through concerted inter-programmatic and inter-sectorial interventions by diverse actors that the heavy impact of human suffering inflicted by this condition will be significantly reduced.

## Competing interest

The author declares that there are no competing interests.
